# Loss to Follow-Up After Pregnancy Among Sub-Saharan Africa-Born Women Living With Human Immunodeficiency Virus in England, Wales and Northern Ireland: Results From a Large National Cohort

**DOI:** 10.1097/OLQ.0000000000000442

**Published:** 2016-04-22

**Authors:** Shema Tariq, Jonathan Elford, Cuong Chau, Clare French, Mario Cortina-Borja, Alison Brown, Valerie Delpech, Pat A. Tookey

**Affiliations:** From the *School of Health Sciences, City University London; †Population and Practice Programme, UCL Institute of Child Health; and ‡Public Health England, London, United Kingdom

**Keywords:** HIV, women, pregnancy, postpartum period

## Abstract

**Background:**

Little is known about retention in human immunodeficiency virus (HIV) care in HIV-positive women after pregnancy in the United Kingdom. We explored the association between loss to follow-up (LTFU) in the year after pregnancy, maternal place of birth and duration of UK residence, in HIV-positive women in England, Wales, and Northern Ireland.

**Methods:**

We analyzed combined data from 2 national data sets: the National Study of HIV in Pregnancy and Childhood; and the Survey of Prevalent HIV Infections Diagnosed, including pregnancies in 2000 to 2009 in women with diagnosed HIV. Logistic regression models were fitted with robust standard errors to estimate adjusted odds ratios (AOR).

**Results:**

Overall, 902 of 7211 (12.5%) women did not access HIV care in the year after pregnancy. Factors associated with LTFU included younger age, last CD4 in pregnancy of 350 cells/μL or greater and detectable HIV viral load at the end of pregnancy (all *P* < 0.001). On multivariable analysis, LTFU was more likely in sub-Saharan Africa-born (SSA-born) women than white UK-born women (AOR, 2.17; 95% confidence interval, 1.50–3.14; *P* < 0.001). The SSA-born women who had migrated to the UK during pregnancy were 3 times more likely than white UK-born women to be lost to follow-up (AOR, 3.19; 95% confidence interval, 1.94–3.23; *P* < 0.001).

**Conclusions:**

One in 8 HIV-positive women in England, Wales, and Northern Ireland did not return for HIV care in the year after pregnancy, with SSA-born women, especially those who migrated to the United Kingdom during pregnancy, at increased risk. Although emigration is a possible explanatory factor, disengagement from care may also play a role.

## Abstract 2

Combining 2 national United Kingdom data sets, we found that 1 in 8 human immunodeficiency virus–positive women were lost to follow-up in the year after pregnancy. This was associated with being Sub-Saharan Africa-born and recent migration.

Between 1200 and 1400 pregnancies are reported in the United Kingdom and Ireland each year among women with diagnosed human immunodeficiency virus (HIV) infection,^1^ most of whom have migrated from sub-Saharan Africa.^2^ The UK and Ireland have a successful program for the prevention of vertical transmission of HIV, with a vertical transmission rate of approximately 0.5%.^3^ Women living with HIV receive antenatal care free of charge, with care usually provided by a multidisciplinary team including specialist staff from HIV medicine, obstetrics and pediatrics, and the voluntary sector.

Sustained engagement with HIV services is important after pregnancy, safeguarding women's health, as well as potentially reducing the risk of unplanned pregnancy and onward transmission of HIV through the provision of contraception and antiretroviral therapy (ART). Discontinuation of ART postpartum has been associated with adverse outcomes, such as detectable viral load at delivery in subsequent pregnancies,^4^ and increased morbidity and mortality.^5^ However, early engagement with HIV services postpartum has been shown to be associated with virologic suppression in the longer term.^6^

Current UK standards of care for people living with HIV state that >95% of patients should access services at least annually.^7^ Studies conducted in the UK looking at loss to follow-up (LTFU) at 1 year among HIV-positive adults have reported rates ranging from 2.5% to 20%, with female sex, younger age, recent diagnosis of HIV, and not being on ART associated with an increased risk of disengagement from care.^8–10^ A consistent finding across these studies is the association between black African ethnicity and LTFU.^8–10^

Women may face particular challenges in engaging with HIV services after pregnancy. However, few studies have explored LTFU after pregnancy in women living with HIV, and most of those have been conducted in low-income settings, revealing high rates of attrition.^11–13^ Data on retention in HIV care after pregnancy in high-resource settings are more limited. Studies in the United States have reported attrition rates of between 40% and 60% 1 year after delivery,^6,14^ whereas a recent analysis of data from the Swiss HIV Cohort Study has revealed a LTFU rate of 12% 1 year postpartum.^15^ To the best of our knowledge, there are no published data on LTFU after pregnancy in women living with HIV in the United Kingdom.

We present one of the largest studies to date in a high-income setting to explore retention in HIV care in women *after* pregnancy. Specifically, we aim to examine the association between LTFU from HIV care in the calendar year after pregnancy and: (i) maternal ethnicity/region of birth and (ii) maternal duration of residence in the United Kingdom, hypothesizing that migrants, especially those living in the United Kingdom for a shorter time, may encounter particular challenges in accessing ongoing HIV care.

## MATERIALS AND METHODS

We present an analysis of data from a matched dataset created from two national HIV datasets in the United Kingdom: the National Study of HIV in Pregnancy and Childhood (NSHPC) and the Survey of Prevalent Infections Diagnosed (SOPHID).

### Data Sources

#### The National Study of HIV in Pregnancy and Childhood

The NSHPC, coordinated at the University College London Institute of Child Health, is a comprehensive population-based active surveillance study of obstetric and pediatric HIV in the United Kingdom and Ireland. Pregnancies in HIV-positive women diagnosed by the time of delivery, and infants born to HIV-positive women, are reported through 2 active parallel schemes managed in collaboration with the Royal College of Obstetricians and Gynaecologists and the British Paediatric Surveillance Unit; full methods are described elsewhere.^16^

#### The Survey of Prevalent HIV Infections Diagnosed

SOPHID is a cross-sectional survey of all individuals aged 15 and above with diagnosed HIV infection who attend for National Health Service (NHS) HIV care in England, Wales, and Northern Ireland (EW&NI) within a calendar year; again, full methods are described elsewhere.^17^ Providers of HIV care submit demographic and clinical data on all individuals who have attended their centre for HIV-related care to Public Health England annually (twice a year in London). Individual data are linked to data from previous years to form a national cohort of persons attending for HIV care.

### Record Linkage

To ascertain whether a woman returned for HIV care anywhere in EW&NI in the calendar year after the end of pregnancy, we created a combined dataset using NSHPC and SOPHID data. Women known by the NSHPC to be pregnant between 2000 and 2009 were matched to the SOPHID data set by year of pregnancy. A hierarchical matching strategy was implemented, using limited identifiers, such as sex, date of birth and residential information. Deaths in women previously reported to the NSHPC were identified through linking of death records held at Public Health England and collated through the Office for National Statistics.

### Eligibility

The analysis was restricted to women with pregnancies reported to the NSHPC with a year of delivery (or estimated date of delivery [EDD] if the outcome was not a live or still birth) between January 2000 and December 2009. During this time, management of HIV in pregnancy in the United Kingdom remained broadly consistent. We only included pregnancies in sub-Saharan Africa-born (SSA-born) and white UK-born women due to small numbers in other ethnic groups. Pregnancies or attendances reported in Ireland and Scotland were excluded from this analysis. The SOPHID does not have data on attendance for HIV care in Ireland. At the time of analysis, Scottish reports to SOPHID before 2008 were not linked over time, and it was therefore difficult to establish links between records on the same patient over time reliably.

### Variables

Maternal ethnicity/region of birth was based on recorded ethnicity and country of birth on NSHPC notification forms and categorized as “sub-Saharan Africa-born” (of black African ethnicity and born in sub-Saharan Africa) and “white UK-born” (of white ethnicity and born in the United Kingdom). Maternal duration of residence in the UK for SSA-born women was based on the length of time in years between reported date of entry to the United Kingdom and estimated date of conception for the reported pregnancy. It was categorized as “>10 years”, “5–10 years,” and “<5 years (before conception).” We hypothesized a priori that women who had migrated to the United Kingdom after conception of the reported pregnancy may be a particularly vulnerable group in terms of access to healthcare and grouped these women separately. Loss to follow-up was defined as no documented attendance for HIV care at an NHS clinic in EW&NI during the calendar year after the confirmed or estimated end of pregnancy.

### Statistical Analysis

Data were analyzed using Stata 11.2 (Stata Corporation, College Station, TX). A χ^2^ test was performed to compare HIV follow-up after pregnancy in women by ethnicity and duration of residence in the United Kingdom. Those who attended for HIV follow-up in the year after pregnancy were compared with those who did not using univariable and multivariable logistic regression models to estimate odds ratios (ORs) and adjusted ORs (AORs), with 95% confidence intervals (95% CI). Year of delivery (or EDD) was included in all final multivariable models as an a priori confounder. Other variables were included if their inclusion significantly improved model fit. This was assessed using likelihood ratio tests, with a significance level of *P* less than 0.05. Sequential pregnancies in the same woman were included in the analysis as independent observations with robust standard errors to account for clustering.^18^

As a sensitivity analysis, we coded all pregnancies that were not matched as lost to follow-up and refitted all multivariable models to explore the effects on estimates.

## RESULTS

There were 8150 eligible pregnancies occurring between January 2000 and December 2009 and reported to the NSHPC by March 2011. Among these, 7219 (88.6%) were in women who had a matched record in the SOPHID database.

Eight women who were known to have died in either the year of pregnancy, or the year after, were excluded from the analysis. This analysis is therefore based on 7211 pregnancies in 5390 women.

### Analysis of Entire Cohort

Demographic and clinical characteristics of the study population are presented in Table 1. Nearly 90% of pregnancies (6485/7211) were in SSA-born women; of whom two thirds had data available on duration of residence in the United Kingdom (4384/6485), with a median of 4.0 years (interquartile range [IQR], 1.7–6.8 years). Just over 10% had lived in the UK for 10 or more years (490/4384), whereas 318 (7.2%) had arrived in the United Kingdom during the reported pregnancy. Most SSA-born women were born in Eastern (3919/6485, 60.4%) or Western Africa (1415/6485, 21.8%).

**TABLE 1:**
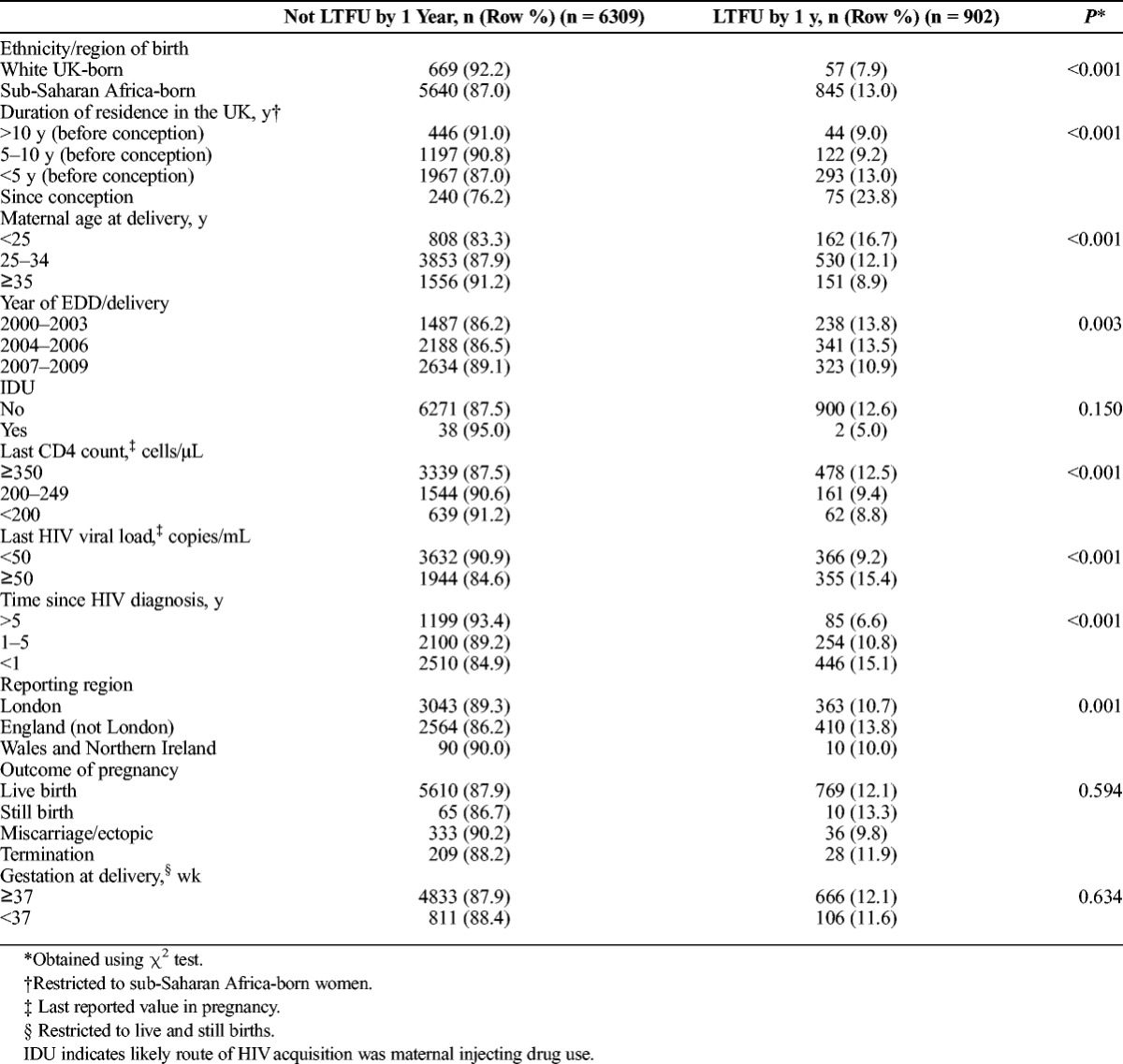
Demographic and Clinical Characteristics of Women With Pregnancies Reported to the NHSPC in 2000–2009 by Maternal Follow-Up in the Calendar Year After Pregnancy

Overall, after 12.5% of pregnancies reported to the NSHPC women did not access HIV care in the year after (902/7211; 95% CI: 11.7%, 13.3%). This proportion rose to 22.5% (1833/8142; 95% CI, 21.6–23.4%) when unmatched records were included and coded as lost to follow-up. Therefore, the proportion of pregnancies where women were lost to follow-up in the subsequent year was likely to be between 12.5% and 22.5%.

**Figure 1:**
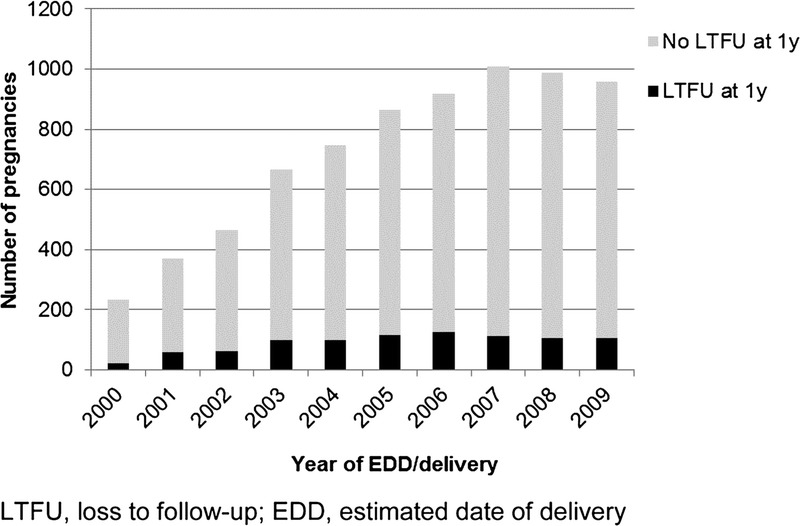
Loss to follow-up in the calendar year following pregnancy, 2000–2009.

The percentage of LTFU varied over time rising from 9.0% in 2000 to a peak of 15.1% in 2003, falling to 11.1% in 2009 (*P* < 0.05; Fig. 1). Of the 902 women who did not return for HIV care in the year after pregnancy, 22.2% returned in the subsequent year. After excluding women who died during the study period, a total of 460 women who had been reported as pregnant to the NSHPC between 2000 and 2009 had not returned for follow-up by the end of 2010 (6.4%; 95% CI, 5.8–6.9%).

Univariable analyses revealed an association between LTFU and younger maternal age at delivery, earlier year of EDD/delivery, higher maternal CD4 count at the end of pregnancy, detectable maternal viral load at the end of pregnancy, more recent maternal HIV diagnosis and pregnancy being reported in areas of England outside London (all *P* < 0.05; Table 1). There was no association between LTFU and either injecting drug use as the route of HIV acquisition or outcome of pregnancy (*P* > 0.1).

A greater proportion of SSA-born women (13.0%; 845/6485) were lost to follow-up compared with women white UK-born women (7.9%; 57/726; *P* < 0.001). After adjusting for maternal age, year, last CD4 count and viral load in pregnancy, time since HIV diagnosis and reporting area, SSA-born women were twice as likely as white UK-born women to be lost to follow-up in the year subsequent to their pregnancy (AOR, 2.17; 95% CI, 1.50–3.14; *P* < 0.001; Table 2). This association persisted but was attenuated on including women who had not been matched across the data sets (AOR, 1.65; 95% CI, 1.27–2.16; *P* < 0.001).

**TABLE 2:**
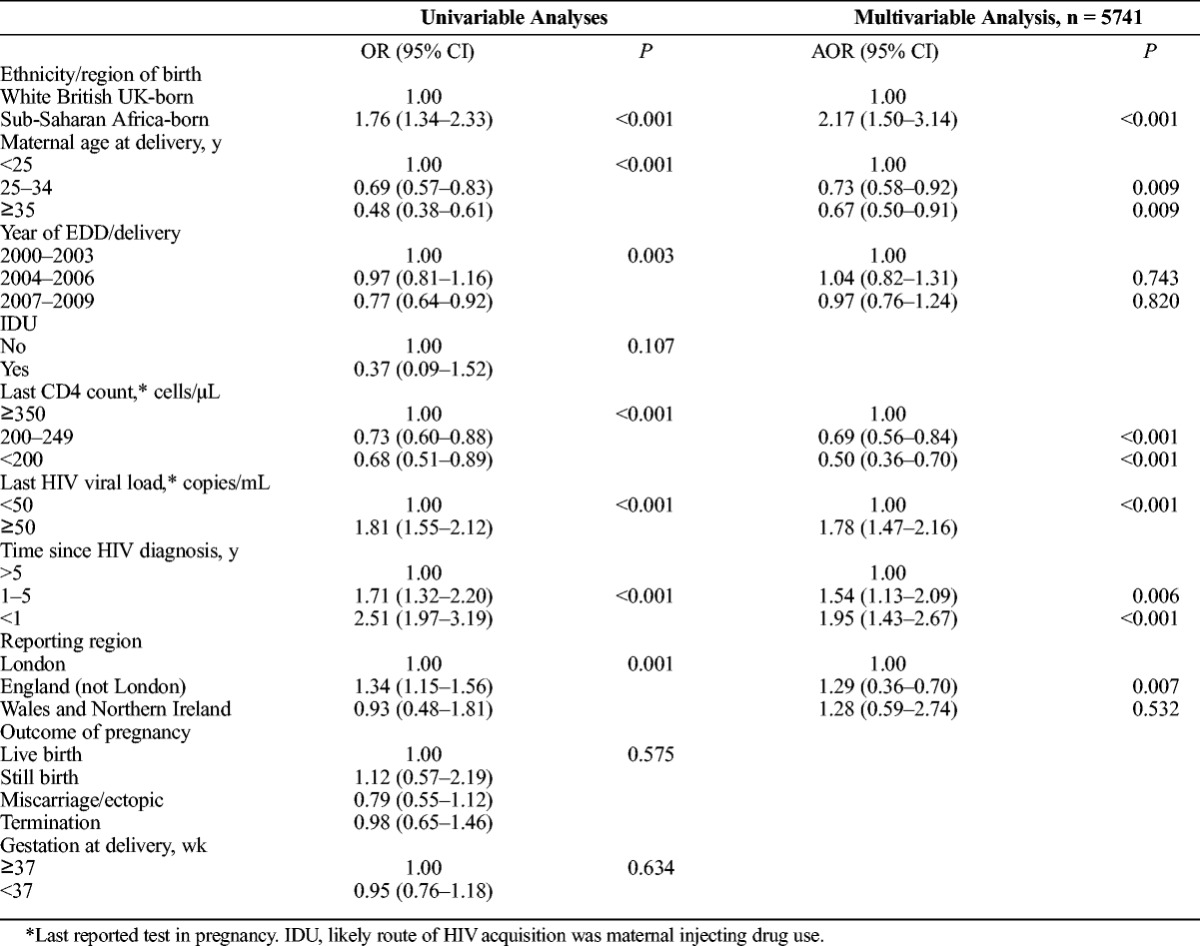
Univariable and Multivariable Analysis of the Association Between Maternal Ethnicity/Region of Birth and Loss to Follow-Up in the Calendar Year After Pregnancy

#### Subgroup Analysis Among Women With Data on Duration of Residence in the United Kingdom

This analysis included 726 white UK-born women, and 4384 SSA-born women who had available data on maternal duration of residence in the United Kingdom. There was a difference in LTFU according to maternal duration of residence in the UK. Among SSA-born women, a greater proportion of those who had arrived in the United Kingdom during the reported pregnancy (23.8%; 75/315), or had arrived less than 5 years before conception (13.0%; 293/2260), did not return for HIV care in the year after pregnancy compared with women who had resided in the United Kingdom for 5 or more years (9.2%; 166/1809; *P* < 0.001; Table 1).

After adjusting for year, last CD4 count and viral load in pregnancy, time since HIV diagnosis and reporting area, the odds of not returning to care in the year after pregnancy were higher for all SSA-born women (regardless of duration of residence in the United Kingdom) compared with white UK-born women (all *P* < 0.01; Table 3). Women who had arrived in the United Kingdom *after* conception were the most likely group to be lost to follow-up (AOR, 3.19; 95% CI, 1.94–3.23; *P* < 0.001). When unmatched women were included in the analysis and coded as lost to follow-up, the odds of LTFU no longer reached statistical significance in SSA-born women who had lived in the United Kingdom for more than 10 years (AOR, 1.34; 95% CI, 0.386–2.10; *P* = 0.201) or between 5 and 10 years (AOR, 1.30; 95% CI, 0.94–1.81; *P =* 0.108). The odds of LTFU were attenuated but remained statistically significant in both SSA-born women who had migrated less than 5 years before their pregnancy (AOR, 1.56; 95% CI, 1.18–2.08; *P* = 0.002) and those who migrated during pregnancy (AOR, 2.27; 95% CI, 1.56–3.29; *P* < 0.001).

**TABLE 3:**
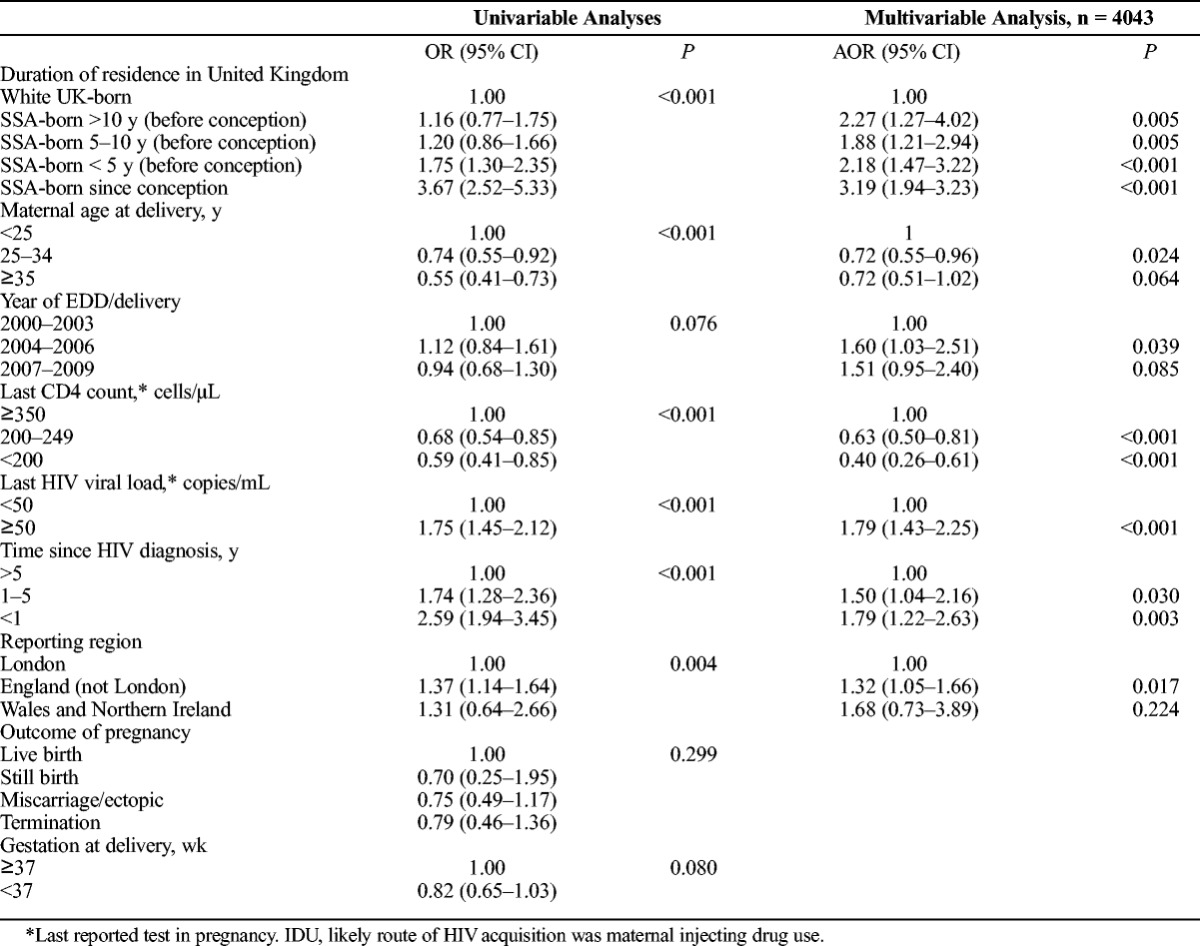
Univariable and Multivariable Analysis of the Association Between Maternal Duration of Residence in the United Kingdom at Conception (in Reported Pregnancy) and Loss to Follow-Up in the Calendar Year After Pregnancy (Restricted to Those With Data on Duration of Residence in the United Kingdom)

## DISCUSSION

In this analysis of 7211 pregnancies occurring in EW&NI during 2000–2009 and reported to the United Kingdom and Ireland's national surveillance programme, we found that 1 in 8 women with diagnosed HIV did not return for HIV care in the calendar year after their pregnancy.

The proportion of women lost to follow-up after pregnancy reported in this article appears to be consistent with data from a previous analysis of UK surveillance data looking at attendance for HIV care in the general HIV-positive population in EW&NI,^8^ but is over twice as high as the rate reported in a recent national audit of LTFU in HIV-positive adults.^10^ Of note, we have also demonstrated a similar LTFU rate as that reported in a recent analysis of data on postnatal retention in care in the Swiss HIV Cohort Study.^15^ Although the proportion of women we identified as lost to follow-up after pregnancy is lower than those described in most studies conducted outside the United Kingdom, it falls short of current UK standards which state that more than 95% of HIV-positive patients should access HIV services at least once a year.^7^ Furthermore, almost 50% of women who did not return for HIV care after pregnancy had a detectable viral load at delivery and were therefore potentially at risk of passing HIV onto any HIV-negative sexual partners. Current UK guidelines recommend offering patients ART regardless of CD4 count to protect their sexual partners and maintain their own health.^19^ Women lost to follow-up will not have benefited from the clinical and public health benefits of this intervention.

Deaths may be underreported or underidentified through linking to the Office for National Statistics; however, they are likely to be few in number and not a major contributory factor to estimates of LTFU. Nearly 90% of women in this analysis had migrated from sub-Saharan Africa, and it is therefore reasonable to assume that emigration may be an important explanatory factor. A recent audit on retention in care in UK HIV services for adults found that 1 in 4 HIV-positive patients not attending for care were known to have left the United Kingdom.^10^ Data on emigration are not available in either the NSPHC or SOPHID datasets, limiting our interpretation.

However, difficulties in accessing care may also play an important role. Factors already known to be associated with disengagement from care after pregnancy in non-UK settings include care-giving responsibilities,^20,21^ suboptimal antenatal care access,^6,12^ institutional barriers,^13,21,22^ maternal substance misuse,^15^ lack of financial resources,^22^ concerns about HIV disclosure and stigma,^12,22,23^ and the lack of support especially from partners.^21,23,24^ Furthermore, women may be highly motivated to engage with care during pregnancy in an effort to secure the health of their baby, but may be less motivated about their own health after delivery.^25^

The overwhelming majority of HIV-positive pregnant women reported during this period were migrants from sub-Saharan Africa, and they were more likely than white UK-born women *not* to attend for HIV care in the year after pregnancy. This is in keeping with other studies that have examined the association between Black African ethnicity and poorer retention in HIV care in the United Kingdom.^8–10^ Furthermore, we observed poorer retention in care among all SSA-born women regardless of duration of residence in the United Kingdom, with those who had migrated during pregnancy being 3 times as likely as white-UK born women to be lost to follow-up. To the best of our knowledge, this is the first study to specifically look at the association between time since migration and retention in HIV care.

Although emigration may be an important explanation for SSA-born women being more likely to be lost to follow-up after pregnancy, it is reasonable to hypothesize that this group may also encounter particular social and structural challenges in accessing HIV services in the long term. Many African migrants living with HIV in the United Kingdom have a high level of social need including financial difficulty,^26^ social isolation^27^ and immigration issues.^28^ Recently arrived migrants may be at particular risk of LTFU due to a complex interplay between insecure immigration status, poverty, and service-related barriers.^29^ It is worth noting that between 2004 and 2012, undocumented migrants and those whose asylum claims had failed were not entitled to free secondary care within the NHS (including antiretroviral therapy outside of pregnancy).^30^

There are limitations in matching records across 2 large national epidemiological datasets which do not share a unique identifier. Incomplete reporting and coding errors in variables would result in a nonmatch and an overestimation of LTFU. Furthermore, inconsistencies in date of birth or residential postcode (which occurs in the context of undocumented migration and highly mobile populations, both groups who are likely to be included in the datasets) would also prevent matching. Although we cannot exclude bias being introduced by non-matches, we are reassured by the lack of evidence of an association between unmatched records and maternal ethnicity/region of birth (*P* > 0.1). Some women who were not matched across the datasets may have disengaged from care after pregnancy leading to possible underestimation of LTFU. Sensitivity analyses showed little effect on ORs in the analysis of ethnicity/region of birth, but did affect estimates in the analysis of duration of residence. However, the associations between LTFU and being resident in the United Kingdom for less than 5 years, although attenuated, remained significant. It is important to acknowledge that we lack information on potentially important confounders such as socioeconomic and immigration status. We also recognize that the category SSA-born elides important heterogeneity; however, we hypothesized that there may be shared experiences of migration and use this as a heuristic device. Finally, these data only reflect attendances for HIV care by the mother and do not capture return for infant care only. We are therefore unable to comment on return for infant testing.

In conclusion, this is one of the largest analyses to date of longitudinal observational data internationally to examine retention in HIV care after pregnancy, and one of very few to be conducted in a high-income setting. It is likely to be representative of experience in EW&NI as the NSHPC and SOPHID both have national coverage and high response rates.^8^ It is reassuring that nearly 90% of women in the United Kingdom with diagnosed HIV infection accessed HIV care in the calendar year after pregnancy. However, 1 in 8 HIV-positive women in EW&NI did *not* return for HIV care in the year after pregnancy, with recently diagnosed women and SSA-born women, especially those who migrated to the United Kingdom during pregnancy, at increased risk. Although emigration is a possible explanatory factor, withdrawal from care may play an important role. It is important for healthcare providers to be aware of the risk of LTFU after pregnancy, especially among women who have been diagnosed recently, those with a detectable viral load at delivery (who may have encountered challenges in adherence and engagement in care), and those from migrant African communities, and to identify culturally sensitive ways of supporting women in accessing long-term HIV care without interruption.
